# Supercharged Fluorescent Protein-Apoferritin Cocrystals for Lighting Applications

**DOI:** 10.1021/acsnano.3c05284

**Published:** 2023-10-30

**Authors:** Marta Patrian, Ahmed Shaukat, Mattia Nieddu, Jesús Agustín Banda-Vázquez, Jaakko V. I. Timonen, Juan Pablo Fuenzalida Werner, Eduardo Anaya-Plaza, Mauri A. Kostiainen, Rubén D. Costa

**Affiliations:** † Chair of Biogenic Functional Materials, 6 Technical University of Munich, Schulgasse, 22, Straubing 94315, Germany; ‡ Department of Bioproducts and Biosystems, Aalto University, 00076 Aalto, Finland; § Department of Applied Physics, Aalto University School of Science, P.O. Box 15100, Espoo FI-02150, Finland

**Keywords:** supercharged fluorescent proteins, protein-cocrystals, luminescent protein cages, color down-conversion, biohybrid lighting

## Abstract

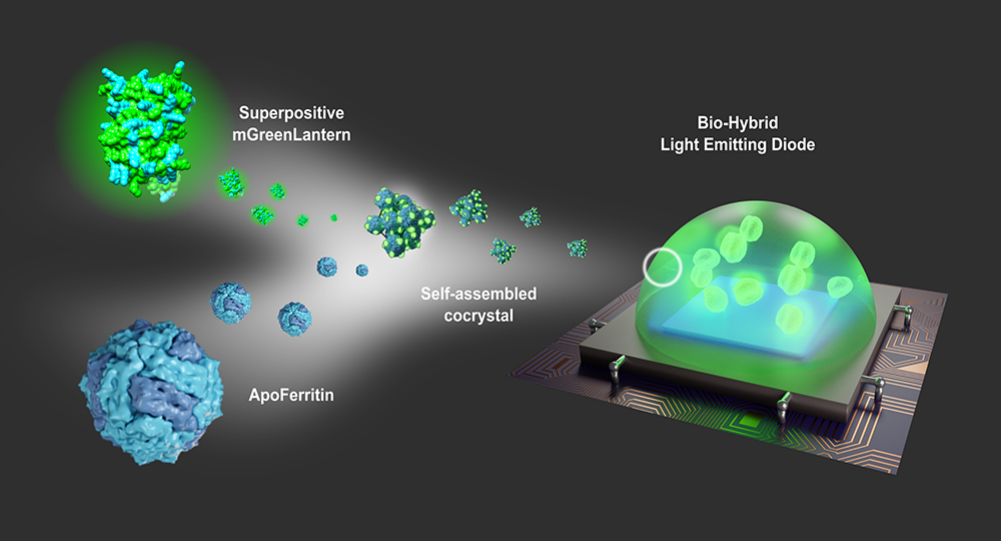

The application of fluorescent proteins (FPs) in optoelectronics is hindered by the need for effective protocols to stabilize them under device preparation and operational conditions. Factors such as high temperatures, irradiation, and organic solvent exposure contribute to the denaturation of FPs, resulting in a low device performance. Herein, we focus on addressing the photoinduced heat generation associated with FP motion and rapid heat transfer. This leads to device temperatures of approximately 65 °C, causing FP-denaturation and a subsequent loss of device functionality. We present a FP stabilization strategy involving the integration of electrostatically self-assembled FP-apoferritin cocrystals within a silicone-based color down-converting filter. Three key achievements characterize this approach: (*i*) an engineering strategy to design positively supercharged FPs (+22) without compromising photoluminescence and thermal stability compared to their native form, (*ii*) a carefully developed crystallization protocol resulting in highly emissive cocrystals that retain the essential photoluminescence features of the FPs, and (*iii*) a strong reduction of the device’s working temperature to 40 °C, leading to a 40-fold increase in Bio-HLEDs stability compared to reference devices.

## Introduction

Though light-emitting diodes (LEDs) will dominate domestic and industrial artificial lighting, their ecoefficient development is a major concern. Major reasons are the lack of efficient recycling protocols and the need for rare-earth-based inorganic phosphors (IPs) as color down-converting filters.^1,2^ In this context, extensive research has focused on hybrid light-emitting diodes (HLEDs), in which organic phosphors based on small molecules,^3^ conjugated polymer,^4^ coordination complexes,^5,6^*etc*. embedded into polymer and epoxy matrices have been explored. Among them, fluorescent proteins (FPs) are considered a paradigm of sustainable development with respect to their cheap bacterial production, easy recyclability, water-processability, and excellent emission merits: high molar extinction coefficients (ε) and photoluminescence quantum yields (ϕ), as well as narrow emission band covering the whole visible spectrum.^7−9^ In addition, FP-based HLED performance stood up with stabilities of >3,000 h and efficiencies of >130 lm/W at low-power conditions^10^ (<50 mW/cm^2^ photon flux excitations), comparable to other devices with traditional OPs: (*i*) perylene diimide-polymer with <700 h@130 lm/W, (*ii*) BODIPYs-polymer with <10 h@13 lm/W, and (*iii*) Iridium(III) complex-polymer with <1,000 h@100 lm/W.^3−6^ In contrast, the device stability is typically reduced to <5 min at high-power operation conditions (blue 200 mW/cm^2^ photon-flux excitation)^11^ due to photoinduced heat generation in the color down-converting coating related to FP motion and efficient heat transfer in a water-rich environment.^10,11^ Several strategies have been proposed to circumvent this issue by (*i*) fabricating waterless polymer composites (<2 h)^10^ and (*ii*) water-free FP isolation using sol–gel chemistry tools (<120 h).^12^

Herein, we explore the hypothesis of whether the immobilization of FPs within a protein cage crystal via electrostatic self-assembly could effectively restrict photoinduced heat generation toward enhanced high-power Bio-HLEDs. Protein cages are self-assembled nanocompartments that are used as ideal building blocks for crystalline assemblies due to their uniform size and shape.^13,14^ As a leading example, apoferritin (aFt) cages feature an inner cavity and crystal pores that can host a myriad of materials: (*i*) ions and metals like Au, Zn, Ce or Ni or Co,^15−19^ (*ii*) organic molecules, such as photosensitizers, host–guest molecules, dendrimers,^20−25^ and (*iii*) biological building blocks, such as protein and peptides.^26−29^ In addition, cocrystallization is driven by electrostatic interactions, resulting in a modular and flexible concept for the formation of aFt-based assemblies with diverse chemical compositions and nanostructures.^30^ However, the cocrystallization with FPs has remained elusive to date. Indeed, we have previously shown that supercharged fusion peptides (i.e., green fluorescent protein (GFP) fused with polylysine peptide) form ordered structures when combined with cowpea chlorotic mottle virus (CCMV).^31^ However, in the case of aFt cocrystals, the presence of GFP hindered the formation of crystalline assemblies, while attempts to engineer super positive GFP variants have led to outstanding thermal stabilities, but at the cost of the ϕ.^32^ This is in line with the design of supercharged enzymes that typically feature an enhanced thermostability, but a reduced activity and/or loss of functionalities.^33,34^

This work answers to these synergistic challenges by disclosing (*i*) engineering approach for the design of a positively supercharged mGreenLantern (scmGL) variant featuring 22 positive charges without losing ϕ, and (*ii*) a protocol to form aFt-scmGL cocrystals that keep the photoluminescence performance of the scmGL. Finally, aFt-scmGL cocrystals were easily implemented in a silicone-based color down-converting filter for high-power Bio-HLEDs, corroborating our initial hypothesis with a strongly reduced photoinduced heat generation from 65 to 40 °C working temperatures. This leads to 40-fold enhanced device stability compared with reference devices.

## Results and Discussion

### Positively Supercharged mGreenLantern

**Figure 1:**
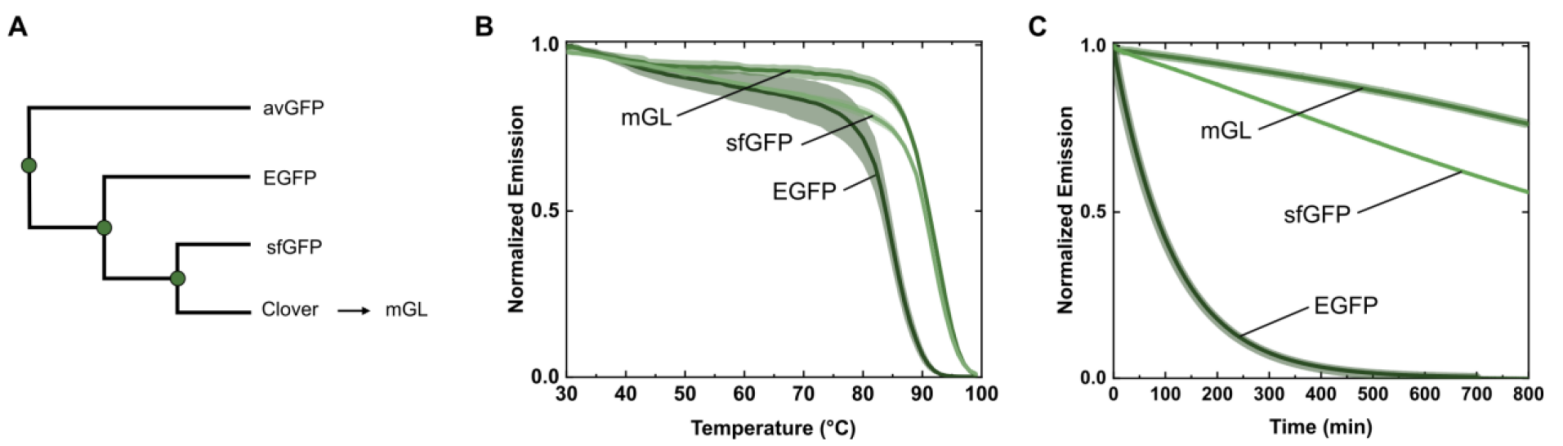
(A) Phylogenetic distances of *Aequorea victoria* avGFP, EGFP, sfGFP, and mGL. (B) *T*_nr_ of mGL, sfGFP, and EGFP in PBS buffer solution. (C) Fluorescence overtime at 70 °C of mGL, sfGFP, and EGFP in PBS buffer solution.

Supercharging often requires extensive surface mutations on the proteins/enzymes, significantly impacting the equilibrium and extent of the noncovalent interactions that allow them to maintain their three-dimensional structure (fold).^32^ To overcome this issue, we first decided to determine the best supercharging starting point among archetypal enhanced green fluorescent protein (EGFP), super folder green fluorescent protein (sfGFP), and the recently published mClover variant called monomeric Green Lantern (mGL) (Figure 1A).^35−37^ To this end, we benchmarked them using the temperature of nonreversibility (*T*_nr_). The *T*_nr_ curves portray irreversible changes in protein structure, leading to changes in fluorescence after exposure to a specific temperature and subsequent cooling.^38^ As shown in Figure 1B, mGL is better compared to EGFP and sfGFP, which both start losing their folding recovery capacity already before 50 °C. At the same time, mGL refolding capacity is only impaired above 80 °C. This results in an increased total area of *T*_nr_ (folding reversibility) going down from 61.8 (mGL), to 58.8 (sfGFP), and to 53.3 (EGFP). To further confirm the higher thermostability showcased by mGL, we measured the fluorescence overtime at 70 °C, mimicking the traditional Bio-HLED operating temperature (Figure 1C).^11^ Here, the superior folding stability of mGL became more prominent, showing a 23% loss after 800 min compared to the 50% and total loss for sfGFP and EGFP, respectively. Thus, we selected mGL as a starting point for supercharging.

The general approach to positively supercharge a protein is based on the identification of polar surface residues, solvent-exposed amino acids (i.e., D, E, N, and Q), under the assumption that these positions will be able to accommodate a positively charged amino acid (i.e., R or K).^32^ A more advanced approach also considers the interaction between surface atoms to preserve the native folding.^39^ However, photoluminescence quantum yield and absorption extinction coefficient (i.e., brightness) in FPs are critically determined by chromophore packing and conformation stability.^40,41^ Thus, we hypothesize that the common brightness loss in supercharged FPs^32,42^ could be related to small changes in the protein fold increasing the free space around the chromophore, reducing its stabilization and brightness. For example, superpositive Cerulean (+32) shows a reduced brightness (Figure S1) and an opening in the barrel near the chromophore of 18.1 Å compared to 17.5 Å of the native Cerulean as noted by X-ray structure Rosetta analysis.^42^ This translates into a higher free volume surrounding the chromophore. In addition, Cerulean +32 shows an almost double conformational flexibility range with respect to wild type (Figure S1). The same pattern is noted in supercharged super folder GFP.^32^ Here, the number of positive charges correlates with the brightness loss, increased free volume surrounding the chromophore, and increased conformational flexibility (Figure S2).

**Figure 2:**
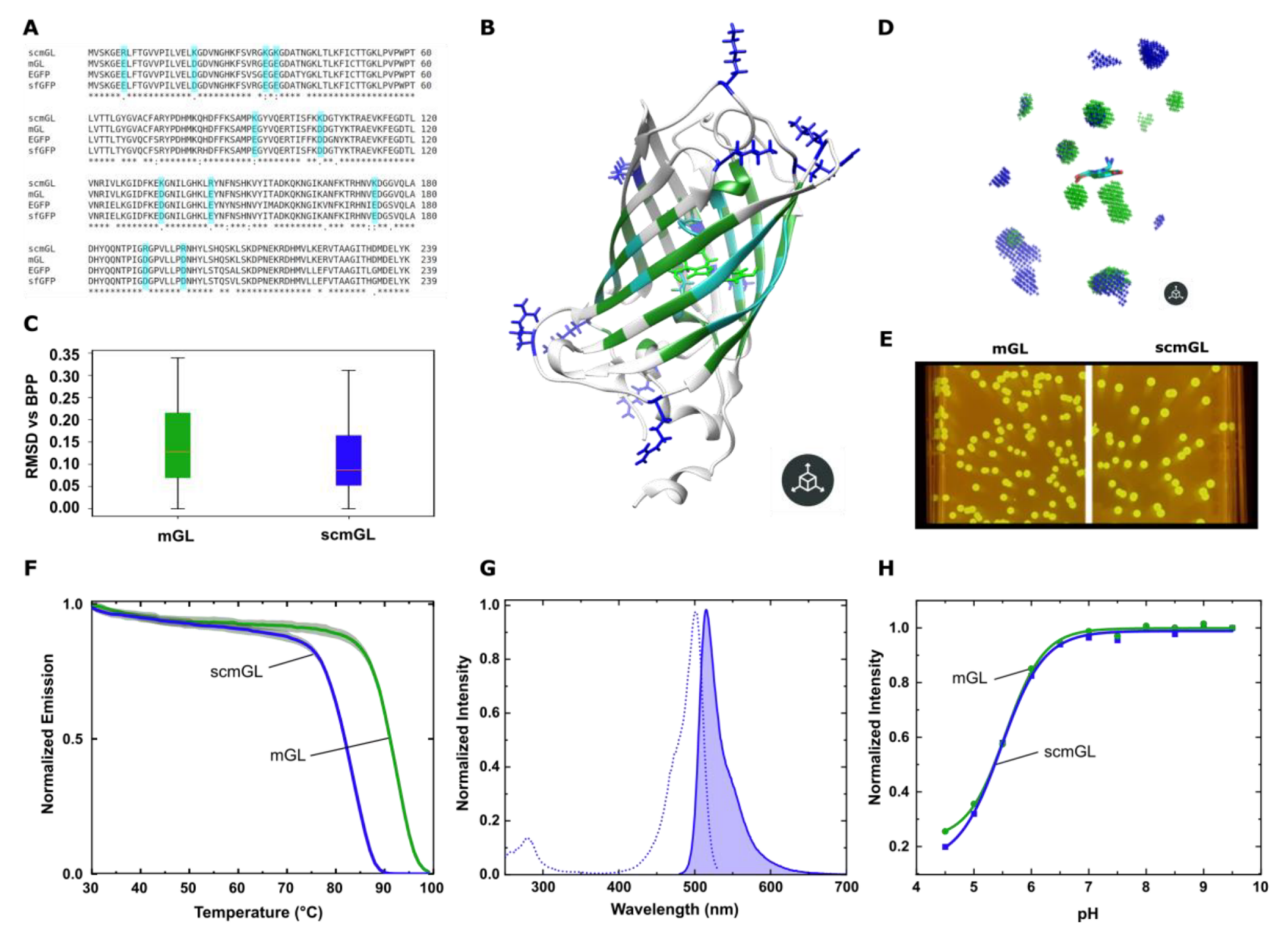
(A) Sequence alignment of mGL and scmGL. (B) Structural representation of scmGL; blue indicates the positively charged mutated amino acids, while dark green indicates the exclusion zone nearby the chromophore with light blue marking the amino acids in direct contact. (C) Root-mean-square deviation (RMSD) analysis of Rosetta minimized populations of mGL and scmGL. (D) Comparison of the internal and peripheral volumes of mGL (green) and scmGL (blue). (E) *E. coli* colonies bearing mGL (left) and scmGL (right) expressing plasmids. (F) *T*_nr_ of mGL and scmGL. (G) Excitation and emission spectra of scmGL (measured at an emission of 520 nm and excitation of 450 nm, respectively). (H) Fluorescence over the pH range 4.5–9.5 of mGL and scmGL.

To reduce this risk, we identified the amino acids near the chromophore based on their van der Waals radii to selectively replace only the polar amino acids that did not interact with any secondary structural elements containing the identified residues above, ensuring that the existing salt bridges remained unaffected (Table S1). Finally, we consider those mutations that are highly exposed to reduce interprotein interaction that might reduce long-range electrostatic interaction in the cocrystallization process.^43^ All-in-all, our strategy leads us to design 11 mutations: eight on the flexible protein loops and three on two β-sheets (Figure 2A,B). This results in a theoretical protein charge of +22 without considering the terminal histidine tag (i.e., +28 in total). Rosetta minimization on AlphaFold models suggested that the mutations have no adverse effect on the protein conformational stability in comparison with mGL (Figure 2C). In contrast, Cerulean and Cerulean +32 exhibited a conformation flexibility range almost double (Figure S1). More importantly, the β-barrel opening at the chromophore site remains constant at 17.5 Å, and our model shows that the free space around the chromophore did not increase after mutations (Figure 2D).

Next, we successfully cloned the genes encoding mGL and scmGL and expressed them in *Escherichia coli*. Both genes resulted in the production of intensely green fluorescent bacteria, as demonstrated in Figure 2E. Subsequently, we purified both proteins using affinity chromatography, achieving similar purity levels, as illustrated in Figure S3. With the purified proteins in hand, we compared the thermal and photophysical characteristics of scmGL to mGL as a reference. First, we observed a decrease in the *T*_nr_ area of scmGL, shifting from 61.8 to 52.8, similar to the *T*_nr_ area of EGFP (Figure 2F). The superpositive character most likely inhibits refolding in the absence of a prokaryotic environment. However, the mutations did not affect its capacity to withstand heat as observed by the similar *T*_m_ (65 °C (scmGL) vs. 66 °C (mGL); Figure S4). On the other hand, excitation and emission spectra strictly resemble those of the parental protein (Figure 2G). In addition, scmGL holds a high ϕ of 70% as that of mGL (72%) along with a 5% lower ε (Table 1). Importantly, scmGL shows still higher ϕ and ε values than sfGFP and EGFP with a brightness of 65.8 vs. 34.1 and 53.9, respectively. Finally, the p*K*_a_ (i.e., pH where only 50% of its chromophore is in an emissive state) of scmGL does not show any variation (Figure 2H and S5), since all the extra ionizable groups are at least 20 Å from the chromophore and primarily located in flexible loops. Consequently, scmGL conserves the broad active pH range of its parent mGL.

**Table 1:** Overview of the Photophysical and Thermal Figures of the Green FPs^a^

protein	λ_abs_ (nm)	ε_max_ (M^–1^ cm^–1^)	λ_ex_/λ_em_ (nm)	Φ (%)	brightness	τ_450_ (ns)	p*K*_a_	*T*_nr_ area	*T*_m_ (°C)
mGL	502	101 800	502/514	72	73.3	3.4	5.4	61.8	66
scmGL	502	94 000	502/514	70	65.8	3.3	5.4	52.8	65
sfGFP	485	83 300	485/510	65	53.9	2.9	5.9	58.8	66
EGFP	488	55 000	488/510	62	34.1	3.0	5.9	53.3	67
aFt-scmGL	502	94 000	502/516	70	65.8	2.8	–	56.3	72

a All excited state lifetime fittings gave errors <0.01.

### aFt-scmGL Cocrystallization

**Figure 3:**
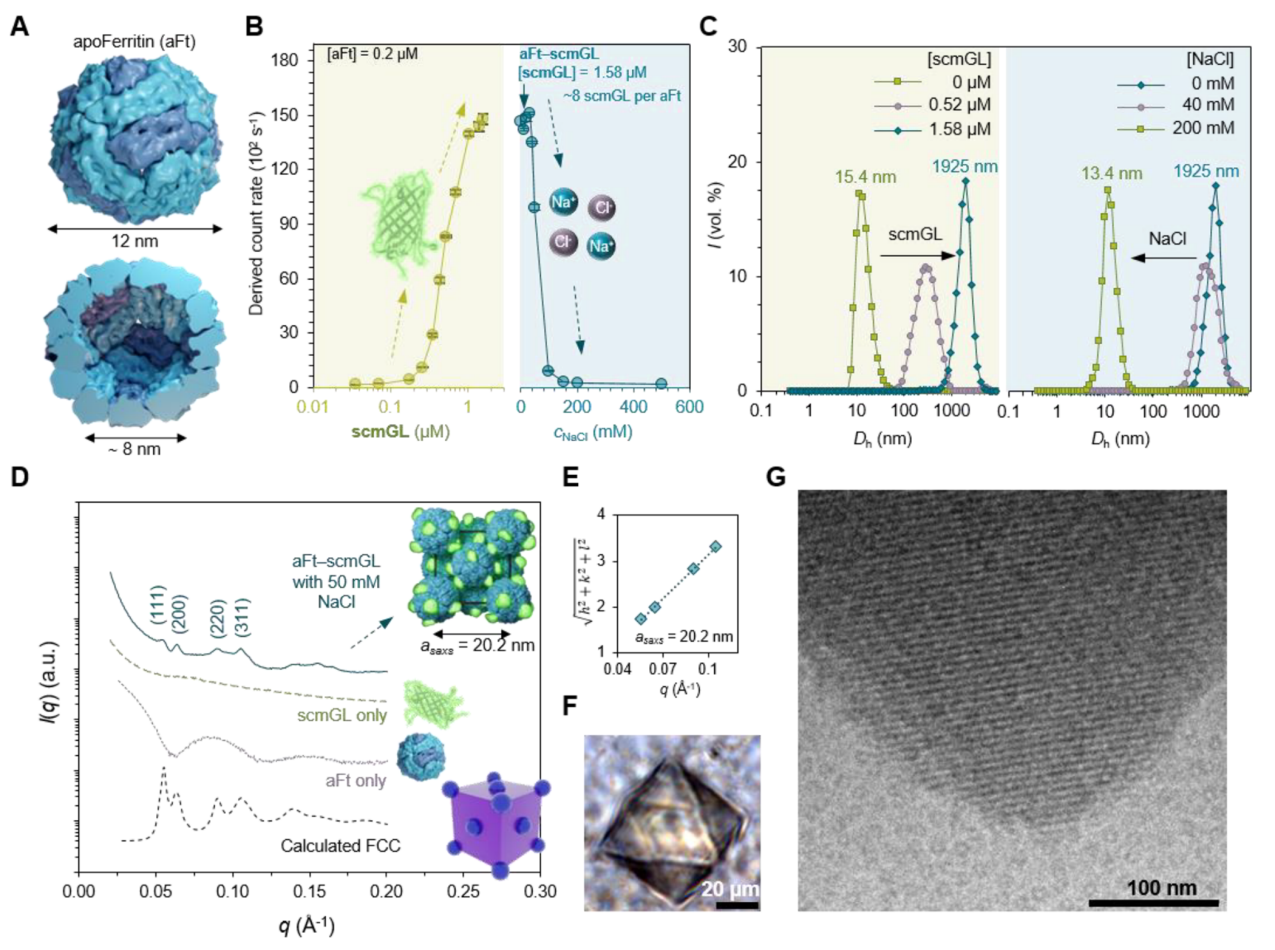
Self-assembly and structural characterization of aFt-scmGL. (A) Structure of the horse spleen apoferritin (aFt; PDB ID: 2W0O). (B) Left: DLS of aFt solution (0.2 μM) titrated with increasing scmGL concentration. Right: The aFt-scmGL complex is disassembled when increasing the ionic strength of the medium with NaCl. (C) DLS data for the volume-averaged size distribution of free aFt titrated with an increasing amount of scmGL (left) and the resulting aFt-scmGL disassembled with NaCl (right). (D) SAXS diffractograms measured for aFt-scmGL complex at 50 mM NaCl, compared to a FCC model, free aFt, and scmGL (offset in the *y*-direction for clarity). Inset: FCC unit cell showing randomly distributed scmGL proteins attached to aFt protein cage. (E) Square root of the sum of the square of the Miller indexes of the assigned reflections for the FCC structure vs the measured *q*-vector positions. Unit cell parameter of *a* = 20.2 nm and space group *Fm*3*m* no. 225. The concentrations in SAXS experiment is aFt 8.3 μM and scmGL 35 μM in 20 mM Tris buffer (pH 7.5). (F) Optical microscopy and (G) cryo-TEM image of vitrified aqueous solutions of the aFt-scmGL complex at 50 mM of NaCl.

The initial investigation of the interaction between aFt protein cages and scmGL involved the application of dynamic light scattering (DLS). This technique enabled the monitoring of changes in the scattering intensity (count rate) and the hydrodynamic diameter (*D*_h_), providing insights into the formation of significant complexes between the two entities. A constant concentration of aFt (0.20 μM, 481.2 kDa) in Tris buffer (20 mM, pH 7.5) was titrated, increasing the scmGL concentration (Figure 3B). At first, aFt shows a rapid increase in the particle count rate, which corresponds to the interaction of these two moieties and the creation of small aggregates. The scmGL amount required to complex aFt fully was 1.58 μM (0.045 mg mL^–1^). Based on the molecular weights of scmGL and aFt protein cages, there are ∼8 scmGL proteins surrounding each aFt protein cage. To establish that the primary driving binding force is electrostatic interactions, the complexes were disassembled by gradually raising the ionic strength of the media, increasing the NaCl concentration (Figure 3B, right),^19^ reaching a total disassembly at 150 mM. The variation in the derived count rate is in good agreement with the change in *D*_h_ that increases with the scmGL concentration (Figure 3C, left). Upon increasing the scmGL concentration, the peak at 15.4 nm (native size of aFt) decreases, and a new peak at ∼2 μm (large aFt-scmGL complexes) evolves at 1.58 μM. At this point, the ionic strength was increased (Figure 3C, right), showing a decrease of *D*_h_ down to a similar size of free aFt (i.e., 13.4 nm at [NaCl] = 200 mM). As a control, the same experimental conditions were applied to prepare cocrystals of mGL and aFt without any success (Figure S6), as expected by the negative surface net charge of mGL (i.e., −2). Thus, our scmGL design, in which the majority of the positive charges are located at the protein loops (Figure 2B), enables strong interaction through long-range electrostatic interactions driving the formation of the assembly.^43^

**Figure 4:**
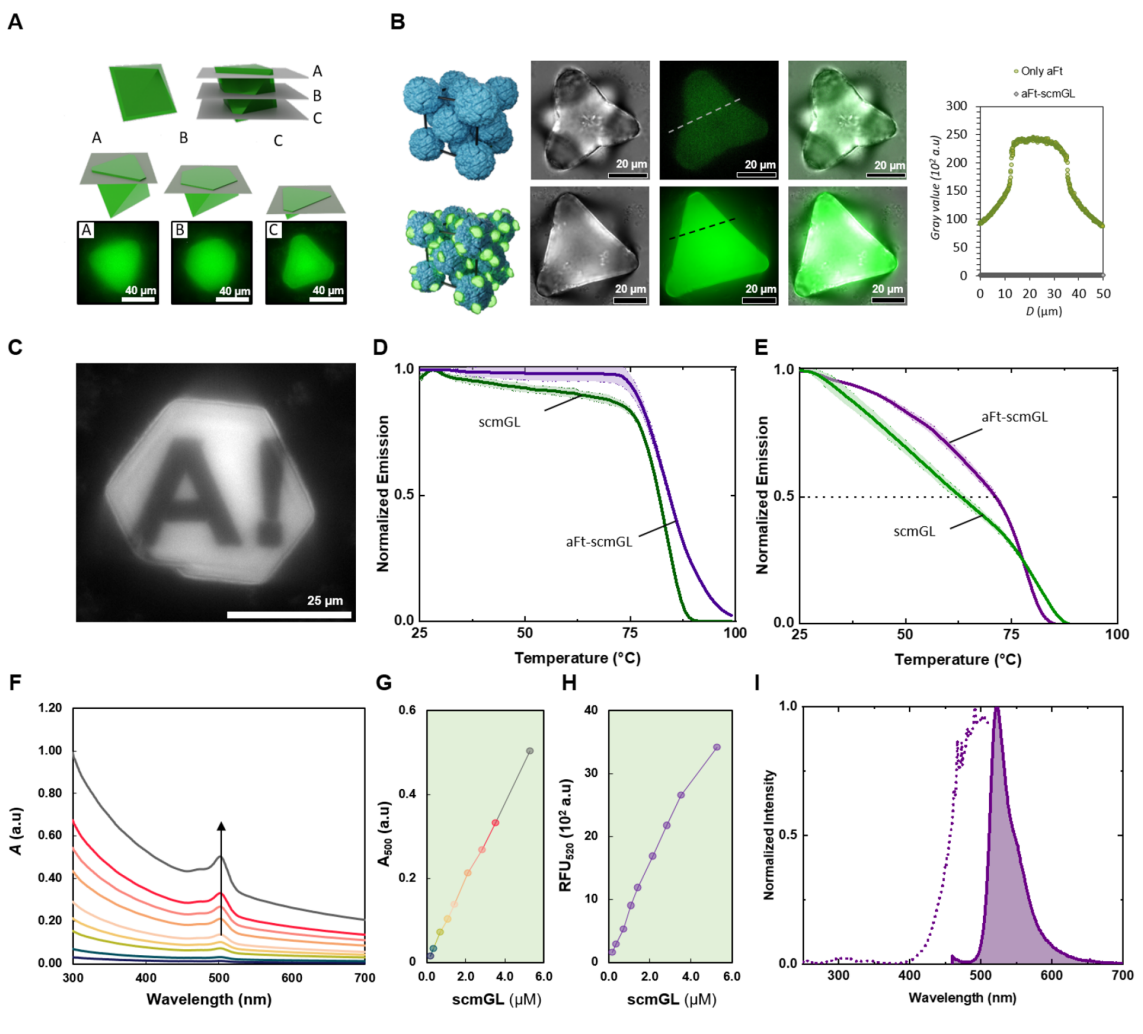
(A) Selected confocal microscopy z-stack planes of a single aFt-scmGL crystal showing that the scmGL is homogeneously distributed in the crystal. (B) Comparison of aFt crystals and aFt-scmGL crystals. Top lane: bright-field, fluorescence, and composite images of the aFt crystals formed using CdSO_4_ [50 mM]. Bottom line: bright-field, fluorescence, and composite images of aFt-scmGL crystals with 50 mM NaCl. Right: Fluorescence intensity profiles (raw pixel values from the sCMOS camera) corresponding to the cross-sectional lines in the fluorescence images (center) of both aFt and aFt-scmGL crystals. (C) Fluorescence microscopy image of a selected area photobleached (Aalto University logo) on the aFt-scmGL crystal, showing that the arrangement of the scmGL in the crystal is static and does not recover when photobleached. (D) *T*_nr_ of scmGL and aFt-scmGL cocrystals. (E) *T*_m_ of scmGL and aFt-scmGL cocrystals. (F) UV–vis spectra of different concentrations of aFt-scmGL cocrystals assuming all scmGL protein is in the crystal. (G) Linear increase in 500 nm wavelength. (H) Linear increase in 520 nm emission wavelength with increase in cocrystals concentration assuming all scmGL protein is in the crystal. (I) Excitation and emission spectra of aFt-scmGL cocrystals (measured at emission of 520 nm and excitation of 450 nm, respectively).

The crystalline structure of aFt-scmGL was determined by small-angle X-ray scattering (SAXS). Protein cages ([aFt] = 8.3 μM = 4 mg mL^–1^) were mixed with scmGL at 35 μM (1 mg mL^–1^) in Tris buffer (20 mM, pH 7.5) at varying NaCl concentrations to facilitate the formation of large assemblies. The SAXS patterns show clear Bragg reflections with well-resolved diffraction peaks at low NaCl concentrations ([NaCl] = 10–50 mM; Figure S7). In line with the above DLS assay, the SAXS pattern gradually faltered at higher NaCl as the assembly formation is hampered (the same SAXS pattern for higher (DLS) ratio observed in Figure S7). The relative peak positions fit with the first allowed reflections of a face-centered cubic (FCC) lattice (Figure 3D). They are assigned to the (111), (200), (220), and (311) reflections, corresponding to ratios *q*_(hkl)_/q* = √3, √4, √8, and √11, respectively. As shown in Figure 3E, a lattice parameter determined for the FCC system is *a*_SAXS_ = 20.2 nm, while the calculated nearest-neighbor aFt center-to-center distance is 14.3 nm. This result is consistent with the diameter of aFt (∼12 nm) and the *D*_h_ obtained from the dynamic light scattering (DLS) analysis mentioned earlier. (15.4 nm; Figure 3C). Moreover, the confirmation of the Bravais lattice space group Fm3m (no. 225) is supported by comparing it to a simulated curve derived from a finite FCC structure. Thus, the FCC unit cell contains four aFt cages corresponding to an estimated amount of *ca*. 32 scmGL proteins per unit cell. Finally, the same octahedral crystal habit is noted for aFt-scmGL and aFt crystals (Figure 3F), suggesting that the [111] direction is thermodynamically favorable for crystal growth. To further confirm the aFt arrangement, cryoelectron microscopy was employed. For the aFt-scmGL sample ([NaCl] = 50 mM) a well-structured organization of individual aFt particles was noted (Figure 3G). To provide evidence of the homogeneous presence of scmGL within the crystals, confocal microscopy was employed, acquiring multiple stacks of images around the focal plane (i.e., z-stack) of single aFt-scmGL crystals (Figure 4A). Here, the distinct planes (top, center, and bottom) illustrate the distribution of scmGL signal throughout the faceted octahedral crystal. Additionally, fluorescence microscopy analysis allowed us to (*i*) a direct comparison of the fluorescence signal between aFt and aFt-scmGL crystals (Figure 4B) and (*ii*) a photobleaching experiment to study the stability of the crystal lattice (Figure 4C). On one hand, the fluorescence signal of aFt-scmGL crystals is 1000 times stronger than the measured background signal of plain aFt crystals formed with the aid of Cd^2+^ salts.^44^ On the other hand, a defined crystal region was photobleached with a high-intensity laser beam (405 nm) without showing any recovery over time, indicating that the excess of unbound scmGL proteins in the surrounding environment did not diffuse and replace the photobleached scmGL proteins within the crystalline assembly. Thus, the aFt-scmGL crystal lattice is a static and stable structure.

Finally, we assessed the thermodynamic and photophysical properties of aFt-scmGL crystals. Concerning the former, the aFt-scmGL shows an increase of the *T*_nr_ area from 52.8 to 56.3 and the *T*_m_ from 63 to 72 °C, surpassing the *T*_m_ of mGL (Figure 4D,E). Thus, the protein packing aids scmGL in retaining/recovering its folding at higher temperatures. The optical properties of the aFt-scmGL cocrystals were examined by using steady-state and time-resolved UV–vis absorption and emission spectroscopy. The UV–vis absorption spectra (Figure 4F,G) display a linear increment of the absorption intensity at 500 nm upon adding more protein cocrystals, as noted for the unbound scmGL protein (Figure S8). Likewise, the aFt-scmGL features a well-structured emission spectrum similar to that of the parent scmGL protein in solution (Figure 4H,I) associated with the same at *ca*. 70% (Table 1). As the only difference, the broadening of the excitation spectrum (Figures 2G and 4I) has been commonly attributed to FP aggregation phenomena that slightly affect the chromophore conformation.^10^ Indeed, the excited state lifetime (τ) is reduced due to the higher refractive index of the neighboring proteins in a multimeric aggregate (Table 1).^45^

### Implementation aFt-scmGL Crystals in Bio-HLEDs

**Figure 5:**
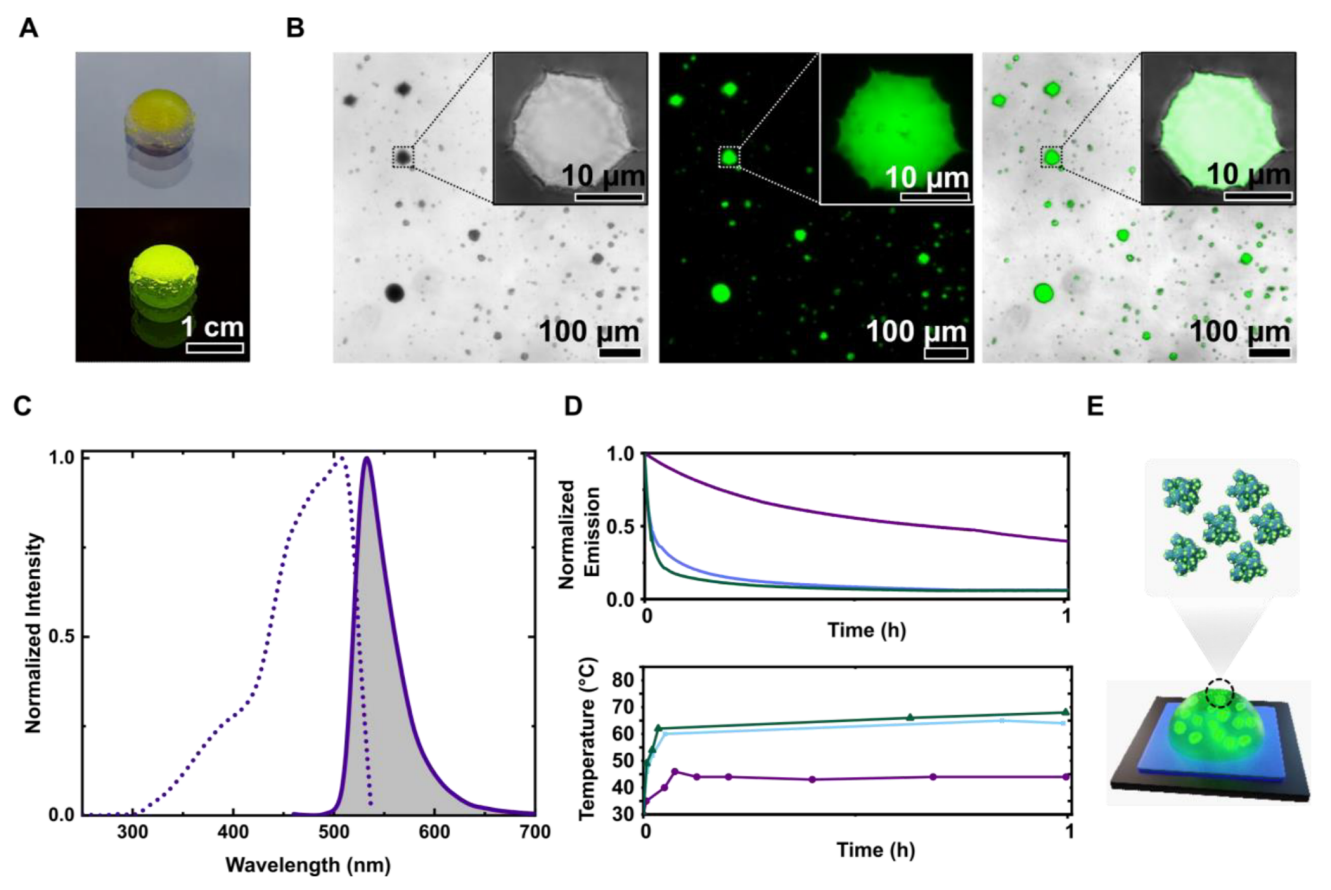
(A) Silicone-based coating with aFt-scmGL cocrystals under room illumination (top) and blue light (bottom). (B) Microscopy images of the aFt-scmGL filter with bright-field (left), confocal fluorescence (middle), and composite (right) images and their respective insets showing magnified image of one of the crystals. (C) Excitation and emission spectra of silicone-based coating with aFt-scmGL cocrystals measured at emission of 520 nm and excitation of 450 nm, respectively. (D) Device stability (top) and temperature (bottom) operating at high power conditions (200 mW/cm^2^). (E) Device schematic of a Bio-HLED showing a blue LED covered by a color-down converting phosphor containing aFt-scmGL crystals.

The most common color down-converting polymer matrix applied to Bio-HLEDs consists of a water-processed mixture of the stabilizer branched poly(ethylene oxide) (PEO) followed by the addition of a large molecular weight PEO to control elastomeric and shape features. This mixture is stirred over a few hours and dried using a gentle vacuum procedure.^7,9−11^ To the naked eye, the aFt-scmGL crystals rapidly dissolve during the first stage. This could be related to synergistic effects: (*i*) disruption of the polymer on the crystalline structure, (*ii*) mechanical stress related to stirring, and (*iii*) change of the local ionic strength due to the final dehydration processes that redissolve the crystals, as demonstrated by the loss in scattering intensity upon addition of PEO (Figure S9). Capitalizing on the effective isolation of cocrystals in highly concentrated solutions, we use a biocompatible and transparent silicone matrix (ELASTOSIL) applied in bioimaging that features a reasonable water-compatibility (<20% v/v) without affecting optical features and a very mild curing process (i.e., room temperature and irradiation free) (Figure 5A). In short, we could combine sedimented aFt-scmGL crystals containing 1 mg of scmGL (37.31 nmol) with 120 μL of ELASTOSIL to obtain dome-shaped down-converting phosphors (Figure 5A). The success of this approach was confirmed by microscopic and photophysical assays (Figure 5B,C and Table 2). Confocal microscopy images confirmed the presence of crystals in which the octahedral shape was mildly rounded (Figure 5B). This is attributed to shear forces during the mixing process and/or minor changes in the local ionic strength. Finally, the shape changes did not affect the fluorescence signal (Figure 5C and Table 2), keeping a green emission band associated with ϕ of 70% and similar τ values to those noted in the cocrystals. However, the excitation spectrum exhibits a pronounced shoulder centered at around 395 nm that is ascribed to a fraction of protonated chromophore.^46,47^

**Table 2:** Overview of mGL, scmGL, and aFt-scmGL Silicone-Based Filters and Their Respective Bio-HLEDs^a^

protein	λ_exc_/λ_em_ (nm)	Φ (%)	τ_450_ (ns)	device stability (min)	device temperature (°C)
mGL	502/527	67	3.3	1	65
scmGL	502/523	70	3.2	1	65
aFt-scmGL	510/530	69	2.7	40	40

a All excited state lifetime fittings gave errors <0.01.

Finally, green-emitting devices were fabricated using a blue-emitting LED chip (450 nm; Winger Electronics, 1W) that was directly covered by an optimized dome-shaped (6 mm ⌀ and 1 mm; Figure 5E) silicone-processed aFt-scmGL (1 mg, 37.31 nmol). At high-power driving conditions (200 mW/cm^2^ photon flux excitation), the device stability (i.e., 50% loss of the initial intensity of the conversion band) was *ca*. 40 min in concert with a working temperature of around 40 °C (Figure 5D). Reference devices with the same amounts of scmGL and mGL showed stabilities of *<*1 min, reaching working temperatures of 65 °C (Figure 5D). The increase in device stability is related to the lack of photoinduced heat generation, as the extensive protein–protein interactions found in aFt-scmGL assemblies (crystalline and amorphous, Figure S10) limit FP motion and heat transfer, as shown by the strongly reduced device working temperature. Importantly, we analyzed the coatings after photobleaching by microscopy and spectroscopic tools, encountering that (*i*) the crystal morphology remains unchanged even in the aftermath of full burnout, and intriguingly, a discernible, though faint, emission signal persists (Figure S11) and (*ii*) a significant increase of the emission intensity at 450 nm related to the chromophore’s neutral form indicating that the primary deactivation mechanism was the photoinduced H-transfer process as noted in other FPs (Figure S12).^10,11^

## Conclusions

This work illustrates an innovative approach in enhancing protein-based Bio-HLEDs device performance with the design of *quasi* zero-thermal quenching color filters with cocrystals of aFt and super positively charged mGL as color down-converting materials. To realize this milestone, three essential advances have been achieved. At first, a design concept for super positively charged FPs, in which peripheral amino acids are mutated, enables to preserve the chromophore environment, leading to a scmGL variant featuring +22 net charge and no loss in thermal and photoluminescence figures in contrast to the prior-art.^32^ In addition, these changes promote efficient long-range electrostatic interactions, resulting in a cocrystal of aFt with FPs. What is more, these cocrystals feature a robust and static assembly with enhanced *T*_nr_ and *T*_m_ in concert with the same ϕ and emission band shape. This allows the fabrication of silicone-based color filters that were applied to on-chip high-power devices. They nicely outperformed the reference ones with a 40-fold enhanced stability due to the significant reduction of the working temperature from 65 °C (FP filters) to 40 °C (aFt-scmGL cocrystals). Overall, this work is seminal, disclosing highly emissive all-protein cocrystals and their earliest integration as active components in energy-related optoelectronics.

## Experimental Section

### Chemicals

Unless otherwise specified, all chemical reagents used in the experiments were obtained from Sigma-Aldrich and were used without any additional purification. The following were also obtained from Sigma-Aldrich: Tris base, horse spleen derived apoferritin (aFt) received in 0.135 M NaCl solution, further diluted in Milli-Q water (aFt, 10 mg mL^–1^). Sodium chloride was obtained from VWR Chemicals. Milli-Q grade water was used in all experiments, while the buffer utilized in all experiments had a pH of 7.5 and consisted of 20 mM Tris base, which was adjusted using 1 M HCl unless otherwise stated.

### FP Production

All constructs used in this study were ordered as codon optimized synthetic genes (Twist Bioscience) cloned within bacterial expression vector pET21(+). All the FPs were under the control of the T7-LacO promoter and N-terminally tagged with 6× HisTag for subsequent protein purification. The FPs plasmid constructs were transformed in electrocompetent *Escherichia coli* BL21 DE3 and plated on Luria Bertani (LB) agar plates, containing 100 μg/mL Ampicillin. Single colonies were picked, inoculated in selective liquid LB medium, and grown shaking overnight at 30 °C. Subsequently, appropriate amounts of preculture were reinoculated in fresh LB to an initial optical density (OD_600_) of 0.1 and grown at 30 °C until OD_600_ 0.6 was reached. At this point, protein production was induced with 1 mM IPTG and the bacterial cells were incubated at 16 °C shaking for 48 h.

### Recombinant Protein Purification

Bacterial cells were harvested via centrifugation (25 min, 5000*g*) and subsequently washed in PBS (NaCl 8 mg/L, KCl 0.2 g/L, Na_2_HPO_4_ 1.42 g/L, KH_2_PO_2_ 0.27 g/L, MQ water; pH = 7.4). The pellets were stored at −20 °C overnight before protein purification. Sonication was performed on ice at 80 Amplitude for a total process time of 8 min. After sonication, the disrupted cell suspension was centrifuged for 1 h at 18 000 rpm. His-tagged proteins present in the supernatant were purified via HisTrap (Äkta pure cytiva) and desalted via HiPrep 26/10 Desalting column (Äkta pure cytiva). Following purification, fluorescent proteins were concentrated with Centrifugal Filter Units (Merck), frozen in liquid nitrogen, and stored at −80 °C.

### Sample Preparation for Cryo-TEM, SAXS, Optical Microscopy, Confocal Microscopy, and UV–Vis

For all the experiments involved complexing the FP and protein cages, the FP proteins were first dialyzed to 20 mM Tris pH 7.5 buffer. The crystals were prepared by first mixing 1.5 μL of 0–800 mM NaCl solution and 1.5 μL of 10 mg mL^–1^ scmGL in a PCR tube and then adding 6 μL 20 mM Tris pH 7.5 buffer and then finally combining 6 μL of aqueous aFt solution (10 mg mL^–1^). The samples were gently mixed with a pipet. Immediately after mixing complexes are formed for 10–70 mM NaCl samples, while samples beyond 80 mM NaCl sample contained no visible complexes. The samples were then incubated in a refrigerator for 24 h to settle down the complexes, and the sediments were used for further characterization. For Cryo-EM, in order to avoid overcrowding of samples on the TEM grid, the samples prepared from the above-mentioned procedure were further diluted three times using a 20 mM Tris pH 7.5 buffer. As a result, the final concentration of aFt in the samples was determined to be 1.33 mg mL^–1^.

### Preparation of FP-Silicone Phosphors

The FP-silicone filters were prepared by mixing 100 μL of ELASTOSIL Part A and 20 μL of ELASTOSIL Part B with 1 mg of aFt-scmGL and their respective mGL and scmGL in 20 mL. The mixture was placed in the desired mold dimensions and dried in air for 3 days to obtain a semispherical coating.

### Device Characterization

The above protein-coatings were placed at zero distance from the 450 nm LED (Winger Electronics; 1 W) or 590 nm LED (Winger Electronics; 1 W). The Bio-HLEDs were characterized using a Keithley 2400 as a current source, while the changes in the electroluminescence spectrum were monitored using an AVS-DESKTOP-USB2 (Avantes) or Avantes Spectrometer 2048L (300 VA grating, 200 μm slit, CCD detector) in conjunction with a calibrated integrating sphere, Avasphere 30-Irrad. The changes in the aFt-scmGL-based coating temperature were monitored using a T430sc thermographic camera (FLIR) coupled to the measuring system. Up to four replicates were measured for each configuration to exclude external influences.

## Supporting Information Available

Supporting results for detailed *in silico* characterization of supercharged fluorescent proteins; protein purification; MSF of green fluorescent proteins; spectroscopic characterization within the pH range 4.5–9; DLS of protein cocrystals; SAXS diffractograms of protein cocrystals at changing NaCl concentrations; absorption and emission of scmGL at changing concentrations; absorption of cocrystals upon addition of polymers; stability of Bio-HLEDs implementing protein cocrystals; microscopy images and emission spectra of photobleached color-down converting filters (PDF)
