# Supplementary material for: Supercharged Fluorescent Protein-Apoferritin Cocrystals for Lighting Applications
Source: ACS Nano. 2023 Oct 30;17(21):21206–15. doi: 10.1021/acsnano.3c05284 (PMC10684032; doi:10.1021/acsnano.3c05284)
Supplement: Supplementary file 1 — nn3c05284_si_001.pdf [file nn3c05284_si_001.pdf]

Supporting Information for

Supercharged Fluorescent Protein-Apoferritin Cocrystals for Lighting Applications

Marta Patrian[a†], Ahmed Shaukat[b†], Mattia Nieddu[a†], Jesús Agustín Banda-Vázquez[a], Jaakko V. I. Timonen[c], Juan Pablo Fuenzalida Werner[a], Eduardo Anaya-Plaza[b]*, Mauri A. Kostiainen[b]*, and Rubén D. Costa[a]**

[a]Chair of Biogenic Functional Materials, 6 Technical University of Munich, Schulgasse, 22, Straubing 94315, Germany. E-mail: jpf.werner@tum.de, ruben.costa@tum.de

[b]Department of Bioproducts and Biosystems, Aalto University, 00076 Aalto, Finland. Email: eduardo.anaya@aalto.fi, mauri.kostiainen@aalto.fi

[c]Department of Applied Physics, Aalto University School of Science, P.O. Box 15100, Espoo FI-02150, Finland

[†]equally contributed

Table of Content

Methods 3

Thermocycler-based modulated scanning fluorimetry 3

Computational methods 3

Dynamic light scattering 3

Cryogenic transmission electron microscopy 3

Small-angle X-ray scattering 3

Optical microscopy 4

Confocal fluorescence microscopy and photobleaching 4

Photophysical characterization of solutions and coatings 4

Supplementary Information – Figure S1 5

Supplementary Information – Figure S2 6

Supplementary Information – Table S1 7

Supplementary Information – Figure S3 8

Supplementary Information – Figure S4 9

Supplementary Information – Figure S5 10

Supplementary Information – Figure S6 11

Supplementary Information – Figure S7 12

Supplementary Information – Figure S8 13

Supplementary Information – Figure S9 14

Supplementary Information – Figure S10 15

Supplementary Information – Figure S11 16

Supplementary Information – Figure S12 17

References 18

Experimental section

Methods

Thermocycler-based modulated scanning fluorimetry

Modulated Scanning Fluorimetry was performed as described in Svilenov *et al.*[1] The Thermocycler CFX96 Touch Real-time PCR System (Bio-Rad) was employed to perform MSF measurements. One standard program composed of heating and cooling cycles ranging from 25 °C to 99 °C was used to measure the progressive loss of fluorescence and the irreversible unfolding of the FPs studied in this work. The samples were heated 5 °C/sec and held for 1 min at the temperature peak, followed by a recovery period of 5 min at 25 °C. Due to the high sensitivity of the Thermocycler detector and high QY of the FPs used in this study, only 1 μM of FPs were added per well to avoid saturation. The thermograms were buffer-subtracted and normalized by the highest fluorescence read-out of each sample. Data analysis was performed using Origin 2019 (OriginLab Corporation, Northampton, MA, USA). Mean values and standard deviations of quintuplicate were calculated and plotted. Melting curves were obtained plotting the fluorescence values obtained at peak temperatures, while non-reversibility curves were obtained plotting the fluorescence values obtained at 25°C. The nonreversibility temperatures (T_{nr}) areas were determined via the integration tool available in the software.

Computational methods

RMSD comparisons: the CA atoms from the conserved glycines in fluorescent protein[2] and the CA from the two flanking residues per chromophore were used for RMSD comparison of every relaxation against the best relaxed structure per protein population.

Cavity detection and Cavity Volume calculations: for Cerulean, Cerulean-32, mGL and scmGL protein structures, the ParKVFinder software[3] was used with default values for whole protein exploration: whole protein mode, low resolution mode, prove in of 1.4 Å, probe out of 4.0 Å, volume cutoff of 5.0 Å^3, and removal distance of 2.4 Å. The volumes in cavities of interest per protein were added for comparison purposes. ParKVFinder usage, along with cavity representations and structure superpositions, were carried out on PyMol 2.5.0.

Dynamic light scattering

The hydrodynamic diameter (D_h) of the assemblies was measured using a Malvern Instruments DLS device (Zetasizer Nano ZS Series) with a 4 mW He-Ne gas laser at a wavelength of 633 nm and an avalanche photodiode detector at an angle of 173°. All experiments were carried at room temperature. PMMA cuvettes were used for the size measurements. Zetasizer software (Malvern Instruments) was used to obtain the particle size distributions: 0.1 mg mL^{-1} of aFt (with a final concentration of 0.25 mM NaCl) dissolved in buffer (20 mM Tris (pH 7.5)) was titrated with different mGL or scmGL concentrations (0.05, 0.1, 1, 10 mg mL^{-1}) to reach the desired ratio (no dilution correction was done as the total addition did not exceed 5 % of sample volume), which was finally titrated with 5 M NaCl to disassemble the complex.

Cryogenic transmission electron microscopy

The cryo-TEM images were collected using JEM 3200FSC field emission microscope (JEOL) operated at 300 kV in bright field mode with an Omega-type zero-loss energy filter. The images were acquired with Gatan Digital Micrograph software while the specimen temperature was maintained at -187 °C. The cryo-TEM samples were prepared by placing 3 μL aqueous dispersion of the sample on a 200-mesh Lacey carbon film on Copper TEM Grids (agar scientific) and plunge-frozen into liquid ethane using Leica grid plunger with 3 s blotting time under 100 % humidity. The grids with vitrified sample solution were maintained at liquid nitrogen temperature and then cryo-transferred to the microscope. The TEM grids were plasma cleaned (20 seconds oxygen plasma flash using a Gatan Solarus). Images were further processed using ImageJ software.

Small-angle X-ray scattering

The SAXS samples were measured using the Xenocs Xeuss 3.0 C device equipped with a GeniX 3D Cu microfocus source (wavelength λ = 1.542 Å) and EIGER2 R 1M hybrid pixel detector at a sample-to-detector

distance of 0.6 m. One-dimensional SAXS data was obtained by azimuthally averaging the 2D scattering data and the magnitude of the scattering vector q is given by $q = 4\pi \sin\theta / \lambda$, where 2θ is the scattering angle. For all the measurements, the scattering vector q was calibrated using a silver behenate standard and the 2D scattering data were converted into SAXS curves by azimuthal averaging. The samples were sealed in 1 or 1.5 mm glass capillaries (Hilgenberg GmbH) that has limited scattering in the measured q region.

Optical microscopy

The Zeiss Axiovert A1 inverted microscope was used to perform imaging through optical microscopy. To prevent distortion of the crystal habit, a chamber-like area was created using double-sided tape on all four sides to hold the sample (3 µL) between the glass slide and coverslip.

Confocal fluorescence microscopy and photobleaching

The confocal fluorescence and brightfield imaging of the crystals was done using a spinning disk confocal microscope (Nikon Ti-E with Crest Optics X-Light V3 scanner and Hamamatsu Orca Flash 4.0LT camera) with photobleaching capabilities (Gataca iLas2) and 60x/1.2W objective lens. The system was controlled using Micro-Manager. Small volumes (3 uL) of the crystal samples (aFt and aFt-scmGL) in buffer were placed between two precision cover glasses (Thorlabs CG15KH) separated from each other using a double sided tape spacer and imaged under identical conditions. Fluorescence images were excited using 470 nm laser (LDI Laser Diode Illuminator) at 1% power level with exposure time of 5 ms. Z-stacks were collected similarly at 500 nm steps. The brightfield images were collected using the same microscope in the transmitted light mode using a red LED (Thorlabs). The selected area photobleaching was done using 405 nm laser (Coherent OBIS 405 nm LX 100mW) at 50% power by raster scanning the beam to form the desired area in ca. 500 ms defined in the software plugin (Gataca Modular).

The confocal fluorescence and brightfield imaging of the silicone based devices (fresh and post-mortem) was done using a point scanning laser confocal microscope (Zeiss LSM710 on Examiner frame). Fluorescence was excited using using 488 nm laser at 2.5% power and emission was collected above 493 nm with 2.5x/0.06 objective lens (low magnification images) and 40x/1.1W objective lens (high magnification images). Fresh sample was the silicone resin mixed with crystals cured on a glass coverslip. The post-morten sample was a bulk piece of moded resin containing crystals.

Photophysical characterization of solutions and coatings

The aFt-scmGL with 50 mM NaCl crystal samples were prepared as described above. The crystals were incubated in the refrigerator for 24 h for sedimenting the crystals. After the incubation, the supernatant was replaced with fresh buffer (20 mM Tris with pH 7.5). For measurements, the UV-Vis and fluorescence spectra were measured using a Cytation 3 plate reader (BioTek) using 96-well plates. Alternatively, absorption spectra were acquired with a UV-vis spectrometer UV-2600 (Shimadzu), using a wavelength range 200–800 nm, scan speed medium, threshold 0.01 and a slit width of 2.0. ε was determined by relative measurement. The molar extinction coefficient was measured using the "alkali-denatured" method,[4] in which scmGL was denatured in 0.5 M NaOH. Under these conditions, the chromophore was converted to a GFP-like chromophore characterized by an extinction coefficient of 44,000 M−1cm−1 at 446 nm. The molar extinction coefficient was calculated based on the absorbance spectra of the denatured and native scmGL. The molecular brightness results from the product of photoluminescence quantum yield and the molar absorption extinction coefficient. The photophysical studies were carried out using a FS5 Spectrofluorometer (Edinburgh Instruments) with the SC-10 module for solid samples, the SC-30 Integrating Sphere to determine ϕ, and the time-correlated single photon-counting or TCSPC (64.3 ps pulse width) module to determine τ. All τ were recorded with an excitation bandpass fixed at 450 nm (τ_{450}) and at the emission maximum. The data was then adjusted to a mono- or bi-exponential decay fit using Origin Software. To calculate the average lifetime for each FP-coating, the following equation was used $< \tau >_0 = \frac{\int_0^x t \sum a_i \, exp\left(-\frac{t}{\tau_i}\right) dt}{\int_0^x \sum a_i \, exp\left(-\frac{t}{\tau_i}\right) dt} = \frac{\sum a_i \tau_i^2}{\sum a_i \tau_i}$; where a_i (λ) is the amplitude fractions and τ_i are the lifetimes. The measurements were performed at room temperature. All the lifetimes recorded were measured with a TCSPC.

Supplementary Information – Figure S1

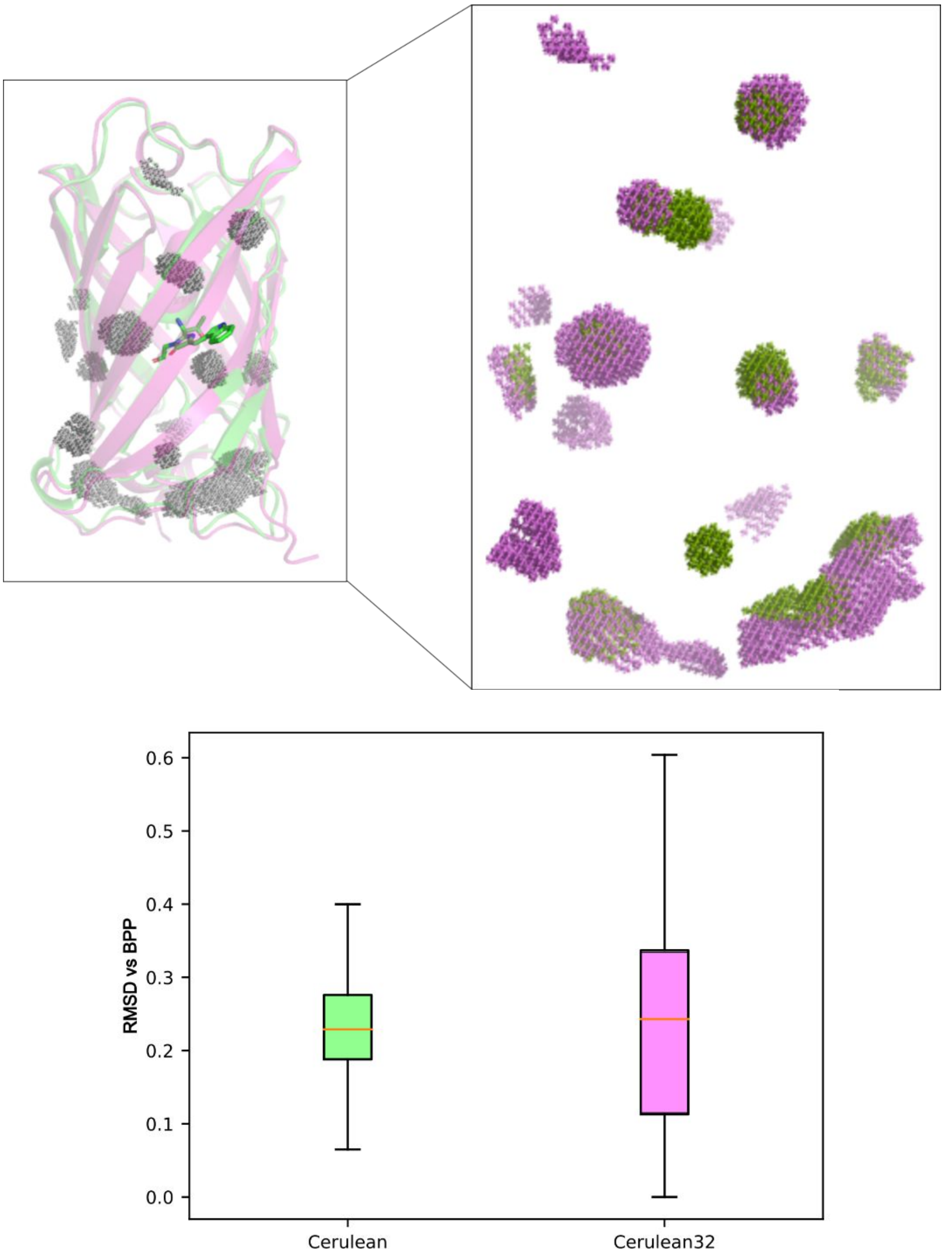

Figure S1. (Top) Cerulean Volume Comparison. Overlay structure super positive fluorescent protein cerulean in violet (PDB: 6MDR:A, net charge +32) and WT Cerulean in green (PDB: 2WSO). Zoom in on the available free volume in the β-barrel structure with the same color code. WT cerulean has significantly less volume available (left), indicating a more compacted structure than the superpositive counterpart. (Bottom) Cerulean and Cerluean +32 root-mean-square deviation (RMSD) analysis of Rosetta minimized populations vs best protein in population (BPP).

Supplementary Information – Figure S2

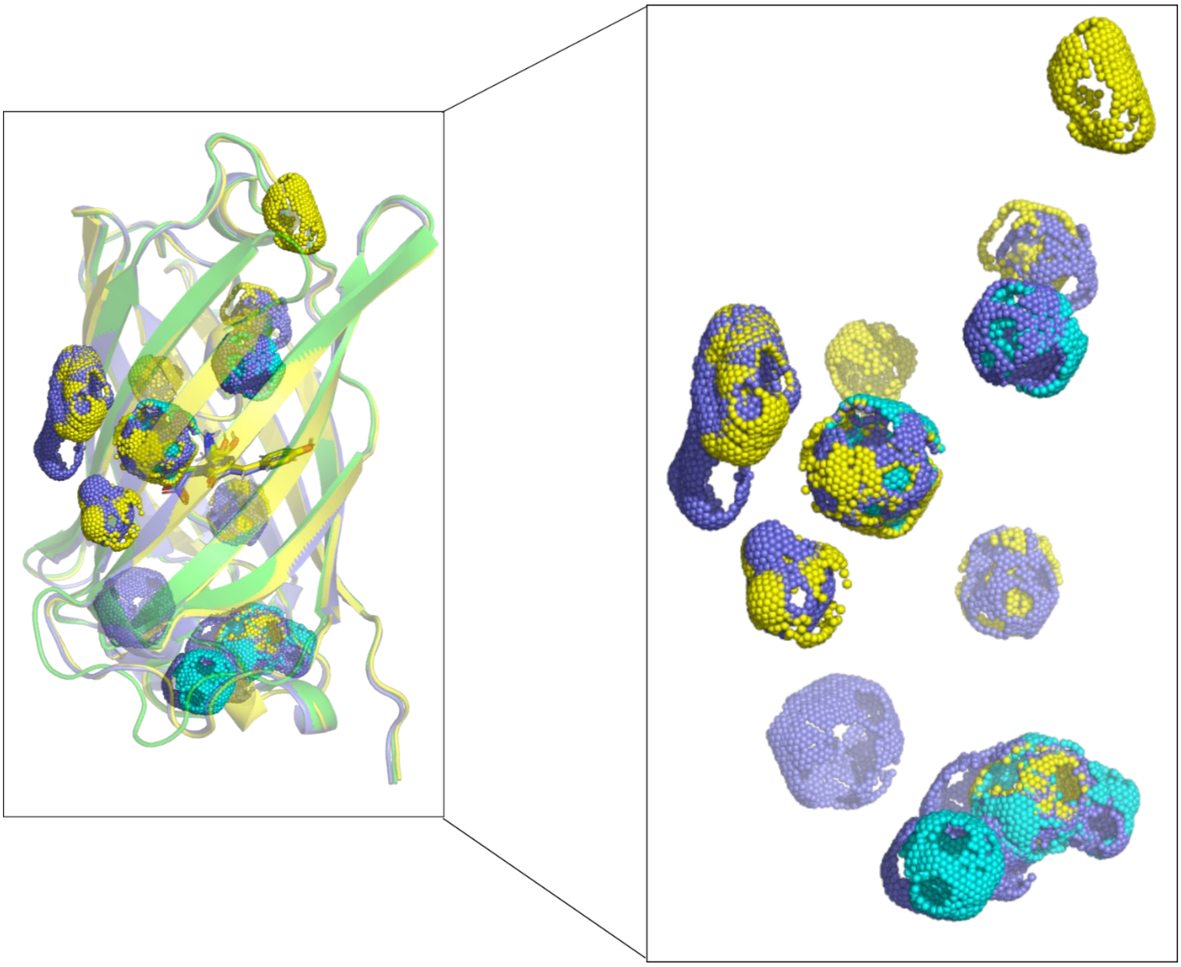

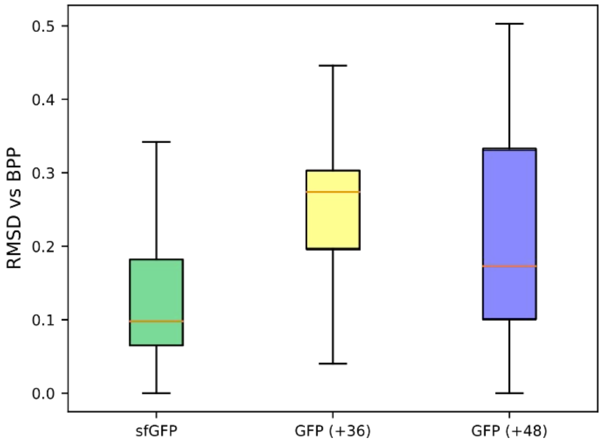

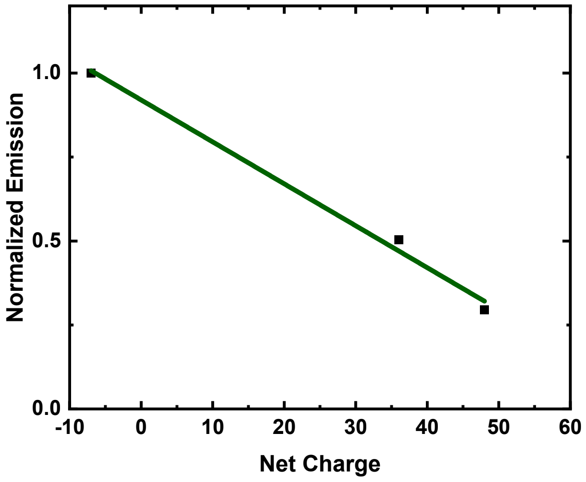

Figure S2. (Top) Overlay structure of sfGFP (green), sfGFP +36 (yellow), and sfGFP +48 (blue). Zoom in on the available free volume in the β-barrel structure with the same color code. (Bottom) sfGFP, sfGFP +36, and sfGFP +48 root-mean-square deviation (RMSD) analysis of Rosetta minimized populations vs best protein in population (BPP) (left) and correlation between net charge of sfGFP and brightness loss (right).

Supplementary Information – Table S1

Table S1. Comparison between H-bonds present on mGL (upper table) and scmGL (bottom table).

mGL

Donor	Acceptor	D-A distance (A)
LYS 79.A NZ	ASP 76.A OD1	3.240
LYS 85.A NZ	ASP 82.A OD2	2.711
ARG 109.A NH1	GLU 111.A OE1	3.085
ARG 122.A NH2	GLU 115.A OE2	2.450
LYS 126.A NZ	ASP 21.A OD2	2.972
LYS 166.A NZ	ASP 180.A OD1	2.309
LYS 166.A NZ	ASP 180.A OD2	2.881
ARG 215.A NE	GLU 213.A OE1	2.945
ARG 215.A NH2	GLU 213.A OE2	3.157

scmGL

Donor	Acceptor	D-A distance (A)
LYS 79.A NZ	ASP 76.A OD1	3.189
LYS 85.A NZ	ASP 82.A OD2	2.794
ARG 109.A NH1	GLU 111.A OE1	3.118
ARG 122.A NH2	GLU 115.A OE2	2.433
LYS 126.A NZ	ASP 21.A OD2	2.915
LYS 166.A NZ	ASP 180.A OD1	2.380
LYS 166.A NZ	ASP 180.A OD2	2.954
ARG 215.A NE	GLU 213.A OE1	2.925
ARG 215.A NH2	GLU 213.A OE2	3.125

Supplementary Information – Figure S3

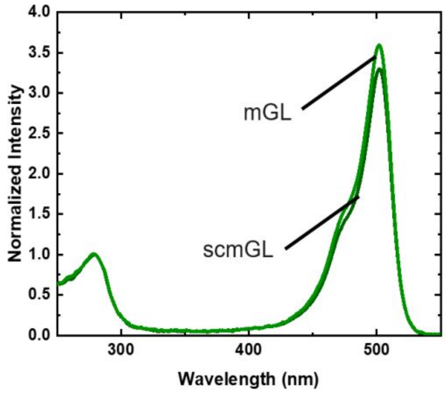

Figure S3. Absorption spectra of mGL and scmGL after affinity chromatography.

Supplementary Information – Figure S4

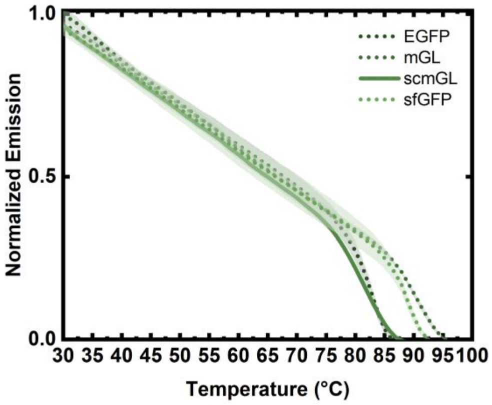

Figure S4. MSF of EGFP, mGL, sfGFP, and scmGL, monitoring the fluorescence intensity upon increasing temperature. T_m is calculated at 50 % of the emission intensity loss.

Supplementary Information – Figure S5

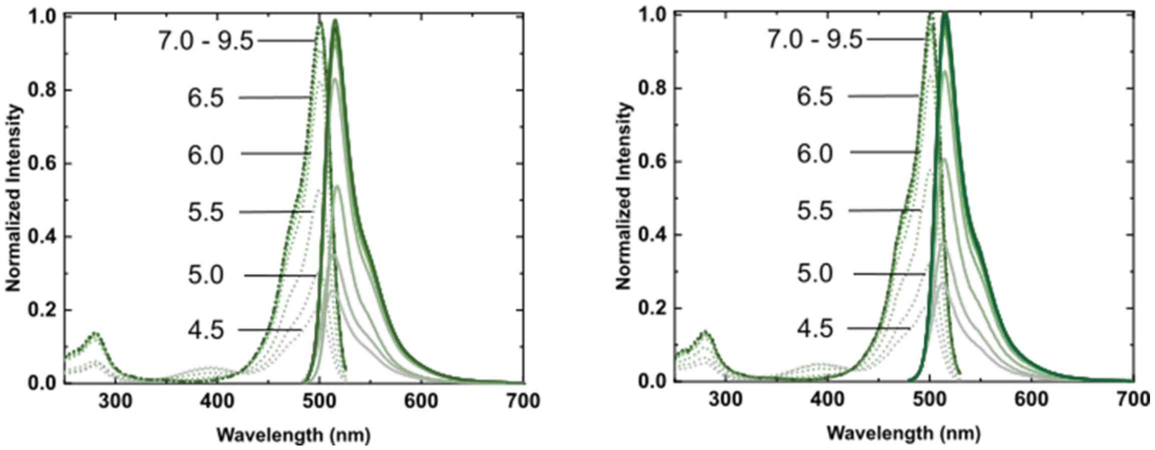

Figure S5. Excitation and emission spectra of mGL (left) and scmGL(right) measured in the pH range from 4.5 to 9.5. In all conditions, excitation was measured at the fixed emission of 520 nm and emission was measured after excitation at 450 nm.

Supplementary Information – Figure S6

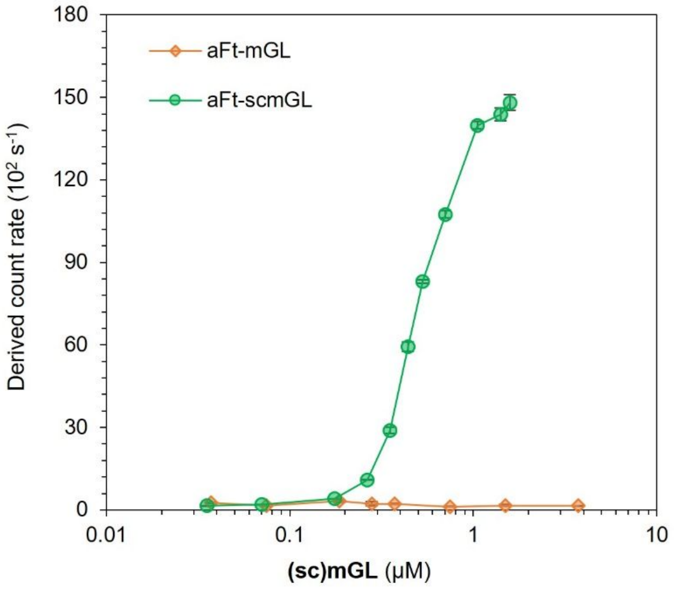

Figure S6. DLS showing the interaction of aFt with mGL with (scmGL) and without (mGL) charged groups.

Supplementary Information – Figure S7

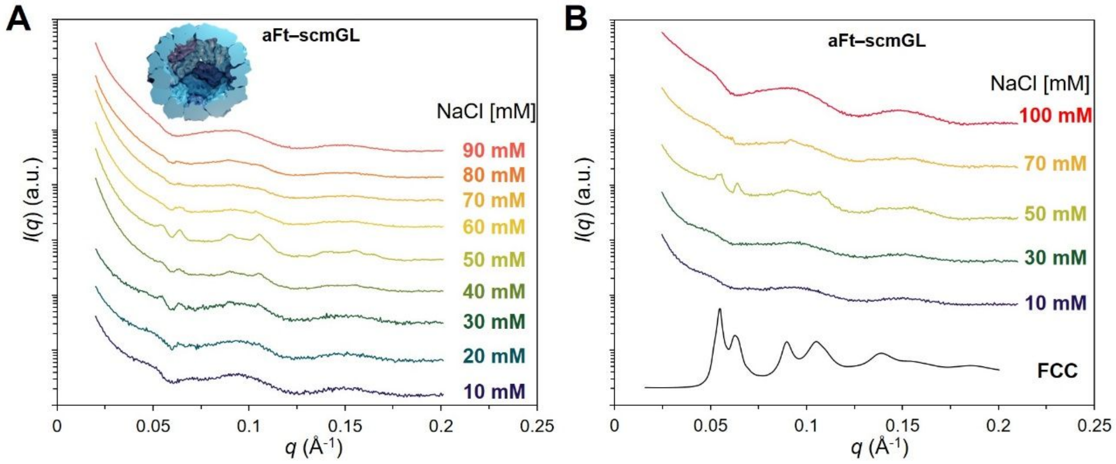

Figure S7. A) SAXS diffractograms of aFt-scmGL changing NaCl concentration ([aFt] = 8.3 μM, [scmGL] = 35,2 μM). B) SAXS diffractograms of aFt-scmGL changing NaCl concentration ([aFt] = 8.3 μM, [scmGL] = 63.3 μM) at DLS ratio.

Supplementary Information – Figure S8

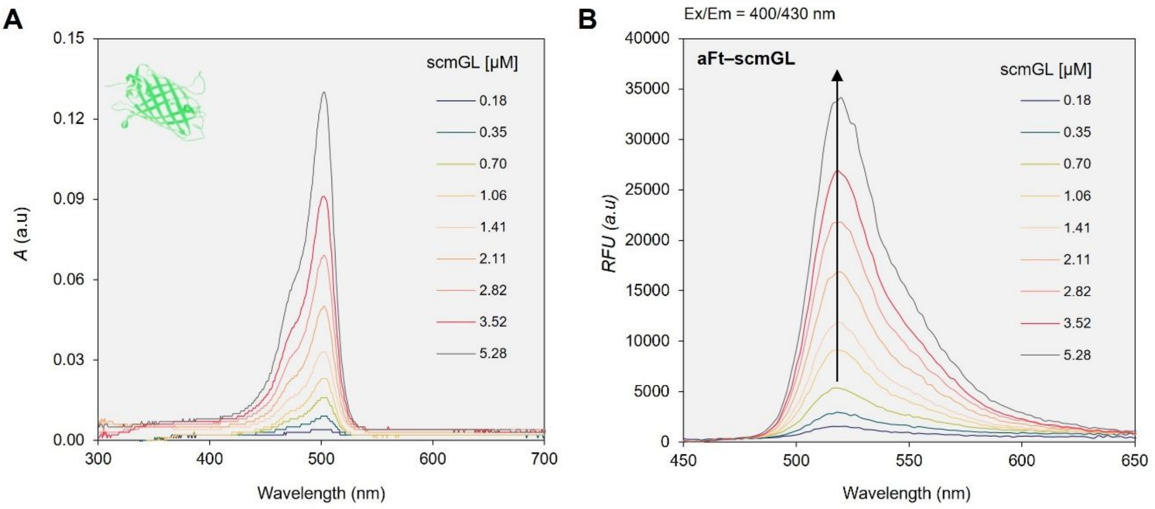

Figure S8. Optical propertise of scmGL UV-Vis absorption spectra of scmGL protein at various concentration ranging from 0.18 to 5.28 µM. B) Fluorescence emission spectra of the same amount of scmGL in crystals. The sample was excited at 400 nm and the emission spectrum was collected from 430 nm.

Supplementary Information – Figure S9

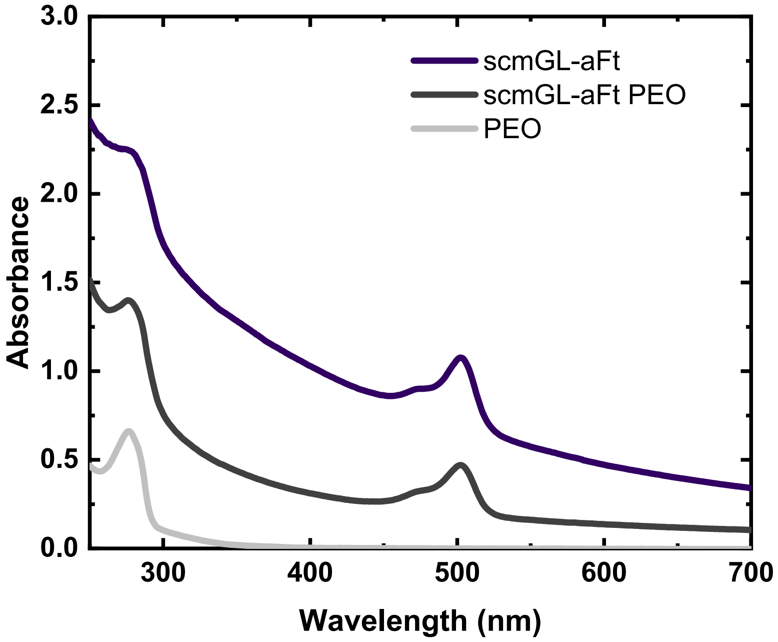

Figure S9. Uv-Vis absorption spectra of scmGL-aFt, scmGL-aFt upon addition of PEO, and PEO.

Supplementary Information – Figure S10

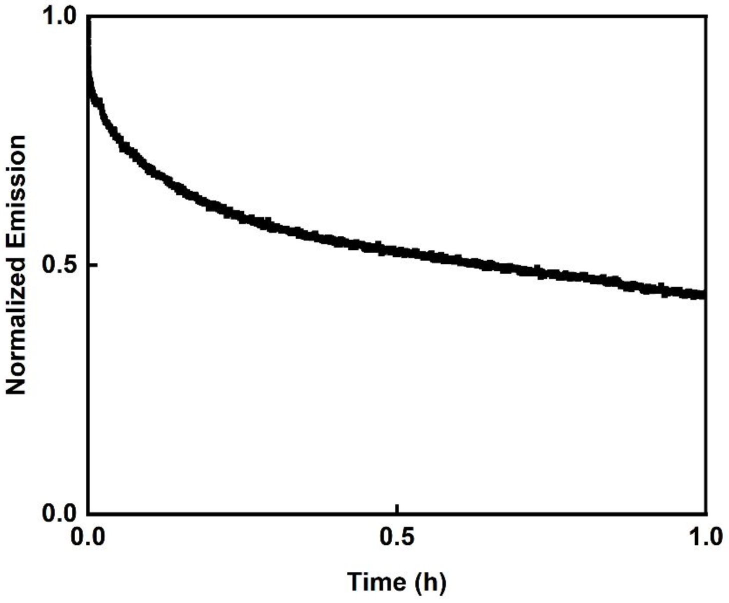

Figure S10. Stability of Bio-HLEDs implementing amorphous aFt-scmGL assemblies operating at high power conditions (200 mW/cm^2).

Supplementary Information – Figure S11

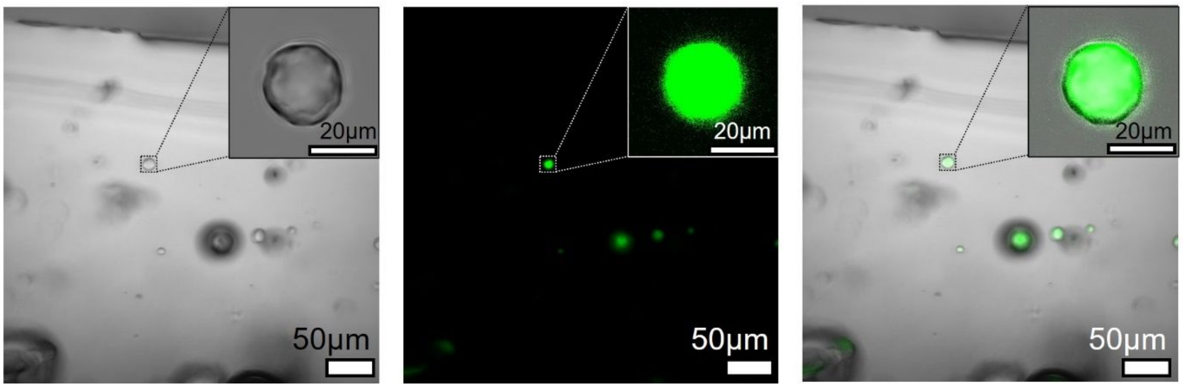

Figure S11. Microscopy image of a photobleached aFt-scmGL-silicone filter with the bright-field (left), confocal fluorescence (middle), and composite (right) images and their respective insets showing magnified image of one of the small crystals.

Supplementary Information – Figure S12

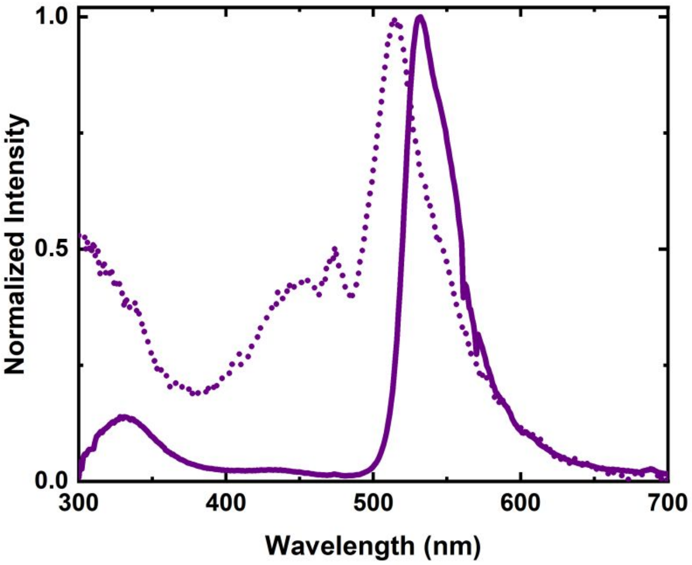

Figure S12. Emission spectra of fresh (solid line) and after photobleaching (dotted line) aFt-scmGL silicone-based coatings at 280 nm excitation.

References

(1) Svilenov, H. L.; Menzen, T.; Richter, K.; Winter, G. Modulated Scanning Fluorimetry Can Quickly Assess Thermal Protein Unfolding Reversibility in Microvolume Samples. *Mol. Pharmaceutics* **2020**, *17* (7), 2638.

(2) Nwafor, J.; Salguero, C.; Welcome, F.; Durmus, S.; Glasser, R. N.; Zimmer, M.; Schneider, T. L. Why Are Gly31, Gly33, and Gly35 Highly Conserved in All Fluorescent Proteins? *Biochem.* **2021**, *60* (49), 3762.

(3) Da Guerra, J. V. S.; Ribeiro Filho, H. V.; Bortot, L. O.; Honorato, R. V.; Pereira, J. G. d. C.; Lopes-de-Oliveira, P. S. ParKVFinder: A thread-level parallel approach in biomolecular cavity detection. *SoftwareX* **2020**, *12*, 100606.

(4) Ward, W. W. Biochemical and physical properties of green fluorescent protein. *Methods Biochem. Anal.* **2006**, *47*, 39.
